# Shotgun metagenomics reveals antibiotic resistome dynamics and metabolic specialization in fungal-dominated microbiomes

**DOI:** 10.3389/fmicb.2025.1626799

**Published:** 2025-12-12

**Authors:** Xin Zhao, Jiedong Qiao, Yongqiang Wang, Hetian Xiong, Runfu Wang, Fenghui Su, Zhuoyi Guo

**Affiliations:** Department of Materials and Chemical Engineering, Taiyuan University, Taiyuan, Shanxi, China

**Keywords:** metagenomics, fungi, microbial genome, antibiotic resistance, metabolism

## Abstract

**Background:**

Metagenomics offers a culture-independent framework for comprehensively characterizing microbial communities by directly extracting and sequencing DNA from environmental samples. In this study, we employed high-throughput metagenomic sequencing to explore microbial communities inhabiting fungal-rich environments, emphasizing taxonomic composition, functional potential, and antibiotic resistance gene (ARG) dynamics.

**Methods:**

Six samples from two distinct groups (HFJ and QFJ) were subjected to Illumina-based shotgun sequencing, followed by rigorous quality control, taxonomic classification, KEGG-based functional annotation, and ARG identification via the CARD database. Comparative analysis revealed stark contrasts between the two groups.

**Results:**

HFJ samples were dominated by eukaryotic taxa, particularly *Saccharomyces cerevisiae*, and exhibited elevated carbohydrate metabolism, aligning with the ecological role of fermentative fungi. Conversely, QFJ samples displayed higher bacterial diversity, particularly Firmicutes and Proteobacteria, and were enriched in lipid and amino acid metabolism pathways. Striking differences were also observed in ARG profiles. QFJ samples harbored greater ARG abundance, particularly genes conferring resistance to beta-lactams, aminoglycosides, and tetracyclines, indicating higher resistance potential and possible horizontal gene transfer activity.

**Conclusion:**

Our results reveal distinct microbial, functional and resistome profiles in fungal-rich versus bacterial-rich fermentation environments. Fungal dominance correlated with lower bacterial diversity and a reduced abundance of certain ARGs, whereas bacterial-rich samples exhibited higher diversity and ARG prevalence. These correlations generate the hypothesis that fungal dominance may suppress bacterial growth or ARG dissemination; however, causal relationships cannot be inferred from our cross-sectional data. The study highlights the potential of metagenomic surveillance to elucidate ecological niches that influence bacterial diversity and resistance dynamics.

## 1 Introduction

Over the last decade, shotgun metagenomic sequencing has become an indispensable, culture-independent approach for profiling microbial communities directly from environmental samples (Aminu et al., 2024). By extracting and sequencing total DNA, researchers can characterize the collective genomes (metagenome) of complex consortia without cultivation, thereby revealing organisms and metabolic pathways that evade traditional microbiology (Lewis et al., 2021; Joseph and Peer, 2021; Benz and Mitra, 2023). Improvements in sequencing depth and bioinformatic pipelines now enable sensitive detection of novel genes, metabolic capabilities, and ecological interactions across soils, fermented foods, and host-associated microbiomes, underscoring the power of metagenomics for ecosystem-level insight (Ariaeenejad et al., 2024; Johnson et al., 2019).

Fermentation ecosystems provide a tractable framework for evaluating how microbial interactions shape community structure and antibiotic resistance. In rice-wine production, molds such as *Rhizopus* spp. and *Aspergillus* spp. secrete *α*-amylase and glucoamylase to saccharify starch, generating fermentable sugars for yeasts including *Saccharomyces cerevisiae* (Keot et al., 2020; Yuan et al., 2024). Non-*Saccharomyces* yeasts such as *Pichia kudriavzevii* contribute aroma complexity and acid tolerance, while lactic acid bacteria modulate flavor and organic-acid profiles (Curiel et al., 2017). Metagenomic surveys of starters and fermented foods have cataloged this diversity and reported high variability of microbial composition and antibiotic resistance genes (ARGs) in some products (Wang et al., 2022). Yet systematic assessments of how fungal versus bacterial dominance modulates community composition, metabolic functions, and resistome dynamics remain limited (Ahmed et al., 2024; Wang et al., 2025).

Fungal-rich environments-ranging from hyphae-dominated soils to post-fermentation matrices-govern organic matter turnover and nutrient cycling, and may also influence ARG distribution and activity (Delgado-Baquerizo et al., 2022; Nazir et al., 2017; Konwar et al., 2024; Piekarska-Radzik et al., 2025). Hyphal networks can transport antimicrobial metabolites and thereby impose selective pressures on neighboring microbes (Keot et al., 2020; Zhang et al., 2025). In rice-wine fermentation, early stages (5–10 days, 20–30 °C, pH 4.0–5.5) feature yeast- and bacteria-driven succession, whereas long fermentations (120–180 days, 10–20 °C, pH 3.5–4.5) favor extensive mycelial mats that could select distinct resistomes (Oh et al., 2024; Hong et al., 2015). These ecological contrasts suggest that fungal dominance might correlate with lower bacterial diversity and reduced ARG abundance, but this hypothesis has not been explicitly tested in fermentation systems.

Here, we applied high-resolution shotgun metagenomics to six rice-wine fermentation mashes representing two production modes: long, natural fermentation with thick fungal mats (HFJ) and rapid, starter-culture fermentation in which mixing suppresses fungal growth (QFJ). We compared (i) microbial community structure, (ii) functional gene repertoires, and (iii) ARG profiles between HFJ and QFJ. We hypothesized that fungal dominance would associate with reduced bacterial diversity and lower ARG abundance due to competitive exclusion and antifungal metabolite exposure. To our knowledge, this work provides one of the first metagenomic comparisons of fungal-rich versus bacterial-rich fermentation ecosystems and offers insights relevant to food safety and antimicrobial-resistance surveillance.

## 2 Materials and methods

### 2.1 Sample collection

In May 2024, six fermentation mash samples were collected from two artisanal rice-wine facilities in Shanxi Province. Both facilities ferment glutinous rice in 500 L open stainless steel tank, but they employ different fermentation regimes. Approximately 5 g of mash was aseptically collected from the upper, active fermentation layer (5–10 cm depth) of each fermenter into sterile tubes, transported on dry ice within 2 h and stored at −80 °C until DNA extraction.

Three stainless steel tank from Facility A underwent long, natural fermentation lasting approximately 120–180 days. During this period thick fungal mats formed on the surface of the mash, and internal conditions stabilized at 10–20 °C with a pH of 3.5–4.5, as is typical of prolonged fermentation phases (Oh et al., 2024). These samples constituted the HFJ group (HFJ1–HFJ3). In contrast, the three stainless steel fermenter from Facility B were subjected to rapid starter-culture fermentation. This process involves an initial saccharification phase of 5–10 days at 20–30 °C and pH 4.0–5.5 followed by vigorous mixing to suppress fungal growth and accelerate fermentation (Steinkraus, 1992; Devanthi and Gkatzionis, 2019). These samples formed the QFJ group (QFJ1–QFJ3). Because each facility contained only three large fermentation stainless steel fermenter in the relevant phase and sampling needed to minimize disruption, we collected a single composite sample from each stainless steel fermenter, yielding three biological replicates per group. Such a design (*n* = 3 per condition) is common in exploratory environmental metagenomic studies where logistical and cost constraints limit replication; for example, an OmicsBox case study of lake microbiomes used three replicates per sampling site and found this sufficient to capture community trends.

This industrial fermentation by-product contains no human or vertebrate material or personal data. Per China’s 2023 Administrative Measures for Bio-project Review, de-identified environmental samples such as these require no ethics approval. Written permission was obtained from both brewery owners, and sampling procedures were designed to minimize contamination.

### 2.2 DNA extraction

Genomic DNA was extracted from each sample using the Qiagen DNeasy PowerSoil Kit (Qiagen, Hilden, Germany), following the manufacturer’s protocol optimized for microbial DNA yield. Briefly, 0.25 g of each sample was subjected to bead beating for mechanical lysis, followed by chemical extraction and purification. DNA integrity was verified via 1% agarose gel electrophoresis, and concentrations were measured using a Qubit 3.0 Fluorometer (Thermo Fisher Scientific, Waltham, MA, USA).

### 2.3 Library preparation, sequencing, and data quality control

Paired-end sequencing libraries were prepared with an insert size of 350 bp (range: 150–150 bp) using the Illumina TruSeq DNA Library Preparation Kit (Illumina, San Diego, CA, USA). DNA was fragmented using a Covaris S220 ultrasonicator, followed by end-repair, A-tailing, and adapter ligation as per the kit instructions. Libraries were amplified via PCR (10 cycles) and quantified using an Agilent Bioanalyzer 2,100 (Agilent Technologies, Santa Clara, CA, USA). Sequencing was performed on an Illumina HiSeq platform, generating paired-end reads of 150 bp each. Sequencing quality was assessed with raw Q20 scores of 97.86–98.05% and Q30 scores of 93.86–94.32%, indicating high-quality output. Raw data were stored as FASTQ files, compressed into.fq.gz format, and organized in a directory structure under 01. RawData/ with subdirectories for each sample (e.g., HFJ1/, QFJ2/) containing paired files (e.g., HFJ1_1.fq.gz and HFJ1_2.fq.gz). Trimmomatic (version 0.39) was used to remove adapters, low-quality bases (Phred score < 20), and reads shorter than 50 bp. Post-filtering, clean read counts ranged from 18,849,303 (QFJ1) to 28,547,218 (QFJ2), retaining 94.84–95.70% of the raw reads. Sequencing quality and retained read percentages for each sample are summarized in Supplementary Table S1. File integrity was verified by computing MD5 checksums for each FASTQ file; the checksum values for all samples are provided in Supplementary Table S4.

### 2.4 Taxonomic annotation

Clean reads were taxonomically classified using Kraken2 (version 2.1.1) with the NCBI RefSeq database, employing default parameters for sensitivity and specificity. Relative abundances were calculated as the proportion of reads assigned to each taxonomic level (kingdom, phylum, class, order, family, genus and species) across samples HFJ1–HFJ3 and QFJ1–QFJ3. Because shotgun metagenomics generates whole-genome fragments rather than amplicons, we did not cluster reads into Operational Taxonomic Units (OTUs), a practice more appropriate for marker-gene surveys. Instead, species-level summaries were derived directly from Kraken2 classifications, enabling the identification of dominant taxa such as *Pichia kudriavzevii* and *Rhizopus arrhizus*.

### 2.5 Functional annotation

Functional profiles were generated by assembling clean reads into contigs using MEGAHIT (version 1.2.9) with default settings. Predicted proteins were annotated against the Kyoto Encyclopedia of Genes and Genomes (KEGG) database using BLASTp (version 2.10.1) with an *e-value* threshold of 1e-5. KEGG Mapper was used to aggregate annotated genes into the top-level functional categories defined by KEGG—Metabolism, Genetic Information Processing, Environmental Information Processing, Cellular Processes, Organismal Systems, Human Diseases and an unclassified group. The sequence number percentages of each category per sample were visualized as stacked bar charts. To identify specific KEGG pathways differentiating the HFJ and QFJ groups, pathway-level counts were normalized and analyzed with LEfSe. LEfSe combines a non-parametric Kruskal–Wallis test with linear discriminant analysis to detect differentially abundant pathways and compute their effect sizes; pathways with (LDA score) > 2 and *p* < 0.05 were considered discriminative. These LDA scores were plotted to highlight group-specific pathway enrichment.

### 2.6 Resistance gene annotation

Antibiotic resistance genes were identified using the Comprehensive Antibiotic Resistance Database (CARD) via the Resistance Gene Identifier (RGI) tool (version 5.1.1). Clean reads were mapped to the CARD database using Bowtie2 (version 2.4.2) with default parameters. Resistance gene abundance was normalized by gene length and sequencing depth (reads per kilobase per million mapped reads, RPKM) and visualized in a heatmap with a phylogenetic tree, constructed using RAxML (version 8.2.12) under the GTRGAMMA model with 1,000 bootstrap replicates.

### 2.7 Statistical and comparative analyses

Alpha diversity (Shannon index) and beta diversity (Bray–Curtis dissimilarity) were calculated using QIIME2 (version 2020.8) to assess microbial richness and community structure differences between HFJ and QFJ groups. Principal Coordinate Analysis (PCoA) was performed on Bray-Curtis distances to visualize sample clustering. Differential abundance of taxa, functional pathways, and resistance genes was tested using DESeq2 (version 1.30.1) with a false discovery rate (FDR) threshold of *p* < 0.05. Visualizations, including stacked bar charts and heatmaps, were generated using R (version 4.0.3) with the packages ggplot2 and pheatmap. Hierarchical clustering in heatmaps was based on Euclidean distance and complete linkage methods.

## 3 Results

### 3.1 Sequencing output and quality control

Six samples—HFJ1, HFJ2, HFJ3 (HFJ group) and QFJ1, QFJ2, QFJ3 (QFJ group)—were subjected to high-throughput sequencing, yielding a range of 19,821,651 reads (QFJ1) to 30,038,531 million (QFJ2) raw paired-end reads per sample. This corresponded to 5.95 to 9.01 gigabases (GB) of sequence data, aligning with common metagenomic data volumes generated by Illumina sequencing platforms and validated using tools such as FastQC for initial quality assessment. Initial read quality was robust, with Q20 scores (percentage of bases with a quality score ≥ 20) ranging from 97.86 to 98.05% and Q30 scores (≥ 30) from 93.86 to 94.32%, values that reflect base call accuracies of 99 and 99.9%, respectively, based on the Phred scoring system (Ewing and Green, 1998). Following adapter trimming and quality filtering using Trimmomatic (Bolger et al., 2014), 94.84–95.7% of reads were retained as clean reads (18.8–28.5 million per sample), with enhanced Q20 (98.85–98.94%) and Q30 (95.99–96.28%) scores. These results confirm the reliability of preprocessing, which is essential for reducing downstream biases in microbial community profiling (Schirmer et al., 2016). Supplementary Figure S1 and Supplementary Table S1 summarizes the sequencing statistics and quality metrics for all samples.

To evaluate assembly completeness, we assembled the cleaned reads using MEGAHIT and assessed contiguity with QUAST. Assemblies contained between 8,500 and 12,300 contigs per library, with N50 values ranging from 780 to 890 bp (Supplementary Table S2). These metrics are comparable to those reported for other environmental metagenomes.

### 3.2 Taxonomic composition of microbial communities

**Figure 1:**
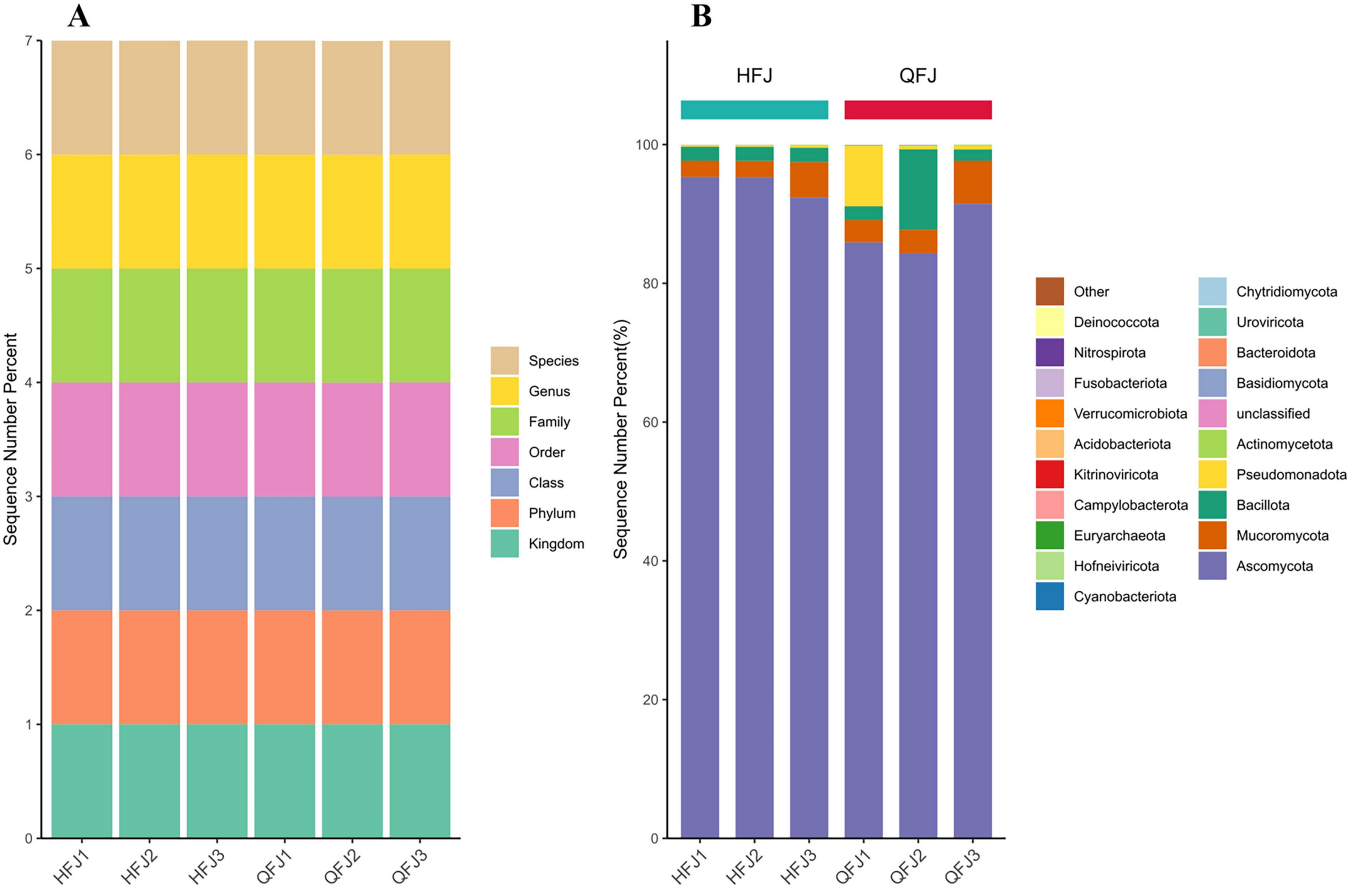
Taxonomic overview of the six metagenomes. **(A)** Depth of read classification achieved with Kraken 2. For every sample, the stacked bar shows the fraction of quality-filtered reads that could be assigned at each taxonomic rank—kingdom through species—demonstrating consistent annotation depth across libraries. **(B)** Phylum-level community structure. Stacked bars give the relative abundance (%) of the 20 most prevalent fungal and bacterial phyla. Ascomycota overwhelmingly dominates all HFJ samples, whereas QFJ libraries contain larger fractions of bacterial phyla such as Bacillota, Actinomycetota and Bacteroidota.

Taxonomic profiling revealed marked differences in microbial community composition between the HFJ and QFJ groups. In the HFJ samples, Eukarya predominated, accounting for 85.36–88.55% of the community, with the yeast *Saccharomyces cerevisiae* being the most abundant taxon (85.32–88.49%), a pattern consistent with fungal-dominated environments where *S. cerevisiae* thrives in nutrient-rich and fermentative conditions. Bacterial taxa were less prevalent (11.45–14.64%), primarily consisting of Firmicutes (10.81–13.88%) and Proteobacteria (0.40–0.59%), which are known to persist in environments with coexisting fungi but at lower dominance (Salvetti et al., 2018). Conversely, QFJ samples displayed a higher bacterial abundance (25.53–32.70%), peaking in QFJ2 (32.70%), with Firmicutes (up to 31.73%) and Proteobacteria (up to 0.72%) as the dominant phyla—groups frequently reported as prevalent in diverse environmental and host-associated microbiomes (Schirmer et al., 2016). Eukarya in QFJ samples ranged from 67.30% (QFJ2) to 93.93% (QFJ3), still driven by *S. cerevisiae* (67.23–93.90%). Figure 1A illustrates the relative abundance of major taxonomic groups at the phylum level across all samples. Because taxonomic ranks in shotgun metagenomics are inherently constant across samples, the stacked bar chart in Figure 1A primarily reflects annotation depth rather than biological differences between HFJ and QFJ. Thus, the near-identical patterns serve as a quality-control check rather than providing mechanistic insight.

At the species level, *S. cerevisiae* was consistently the most abundant across all samples. Other notable species included *Fructilactobacillus fructivorans* (enriched in HFJ samples), a microbe commonly observed in fermentation-associated ecosystems such as wine and coffee production (Góngora et al., 2024), and *Acinetobacter soli* (elevated in QFJ1), which has been identified in soil and clinical settings and is recognized for its environmental adaptability (Akter et al., 2021). Additional species, such as *Pichia kudriavzevii* and *Rhizopus arrhizus*, were detected at low but measurable abundance. Quantitative analysis of the Kraken2 read counts showed that *P. kudriavzevii* comprised between 1.17 and 1.56% of total reads in HFJ samples and 1.38 to 3.03% in QFJ samples, with mean relative abundances of 1.43% (HFJ) and 1.96% (QFJ). *R. arrhizus* was rarer, accounting for 0.03–0.08% of reads in HFJ samples and 0.04–0.10% in QFJ samples (mean 0.06% in both groups; see Supplementary Table S3). A Mann–Whitney U test indicated no significant differences between groups for either species (*p* > 0.5). A stacked bar chart of the top species by relative abundance is presented in Figure 1B, and a supplementary plot summarizing the relative abundances of *P. kudriavzevii* and *R. arrhizus* across all samples is provided in Supplementary Figure S2.

### 3.3 Functional profiles of microbial communities

**Figure 2:**
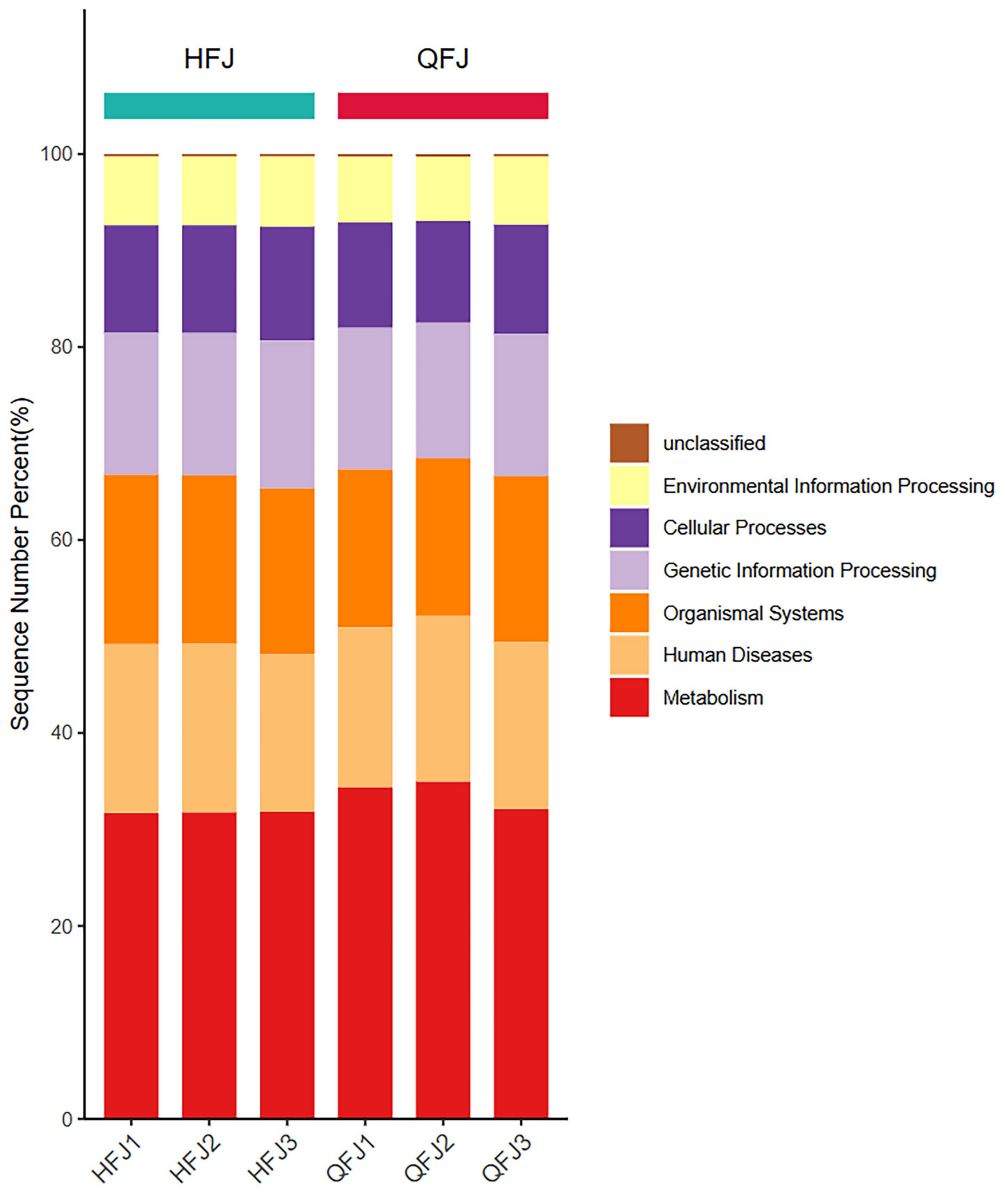
KEGG functional category distribution across samples. Sequence-level KEGG functional composition of HFJ and QFJ libraries. Stacked bars represent the percentage of reads assigned to each top-level KEGG category (Metabolism, Human Diseases, Organismal Systems, Genetic Information Processing, Cellular Processes, Environmental Information Processing and unclassified).

KEGG annotation showed that the majority of predicted genes in all six libraries belonged to the Metabolism category (∼30–35%), followed by Human Diseases and Organismal Systems, while Genetic Information Processing, Cellular Processes, Environmental Information Processing and unclassified functions contributed smaller proportions (Figure 2). This overall distribution of top-level KEGG categories was similar across HFJ and QFJ samples, indicating comparable high-level functional potential in both groups.

**Figure 3:**
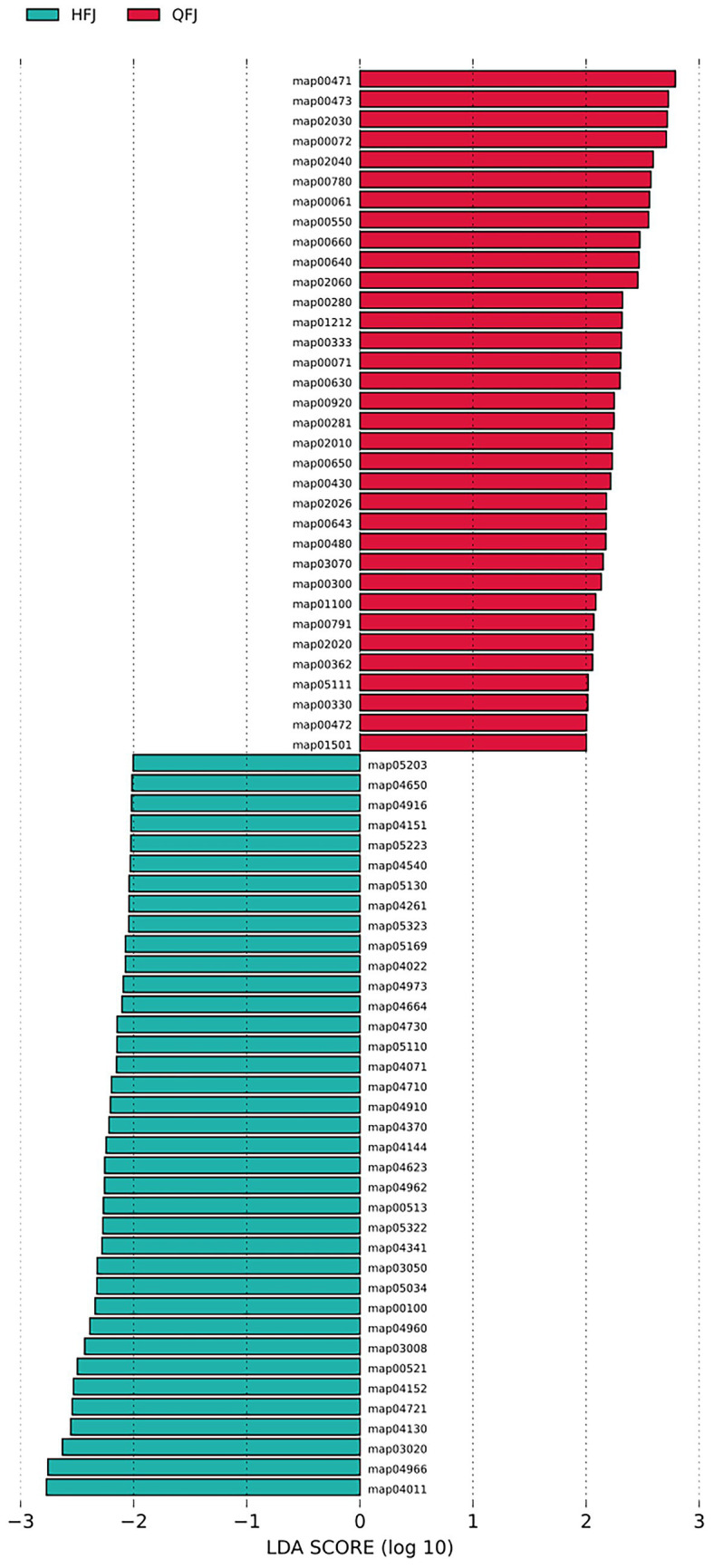
KEGG pathways differentiating HFJ and QFJ samples identified by LEfSe. Linear discriminant analysis (LDA) scores of KEGG pathways distinguishing the HFJ and QFJ groups. Each horizontal bar corresponds to a KEGG pathway, plotted according to its log^₁₀^(LDA score). Bars extending to the right (crimson) indicate pathways enriched in QFJ samples, whereas bars extending to the left (teal) indicate pathways enriched in HFJ samples. Only pathways with *p* < 0.05 and |LDA score| > 2 are shown; the length of each bar reflects the effect size estimated by LEfSe.

Differential pathway analysis identified numerous KEGG pathways with significant differences between HFJ and QFJ. LEfSe detected 71 pathways with |LDA score| > 2; positive scores (crimson bars) correspond to pathways enriched in QFJ and negative scores (teal bars) to pathways enriched in HFJ. The majority of discriminative pathways were enriched in QFJ, while a smaller number were more abundant in HFJ. Figure 3 presents the LDA scores (log^₁₀^ scale) for these pathways, illustrating the functional distinctions between the two groups.

### 3.4 Antibiotic resistance gene profiles

Analysis of antibiotic resistance genes (ARGs) using the Comprehensive Antibiotic Resistance Database (CARD) revealed clear differences in the resistome profiles of the HFJ and QFJ groups. CARD, a curated resource designed for precise identification and classification of resistance determinants, enabled the annotation of ARGs directly from metagenomic sequences using its model-based framework (Jia et al., 2016). The HFJ samples exhibited lower ARG abundance and diversity, whereas QFJ samples were enriched in genes conferring resistance to beta-lactams, aminoglycosides, and tetracyclines—three antibiotic classes known to dominate environmental and clinical resistomes globally (Napit et al., 2025). This trend suggests greater potential for resistance dissemination in QFJ environments.

**Figure 4:**
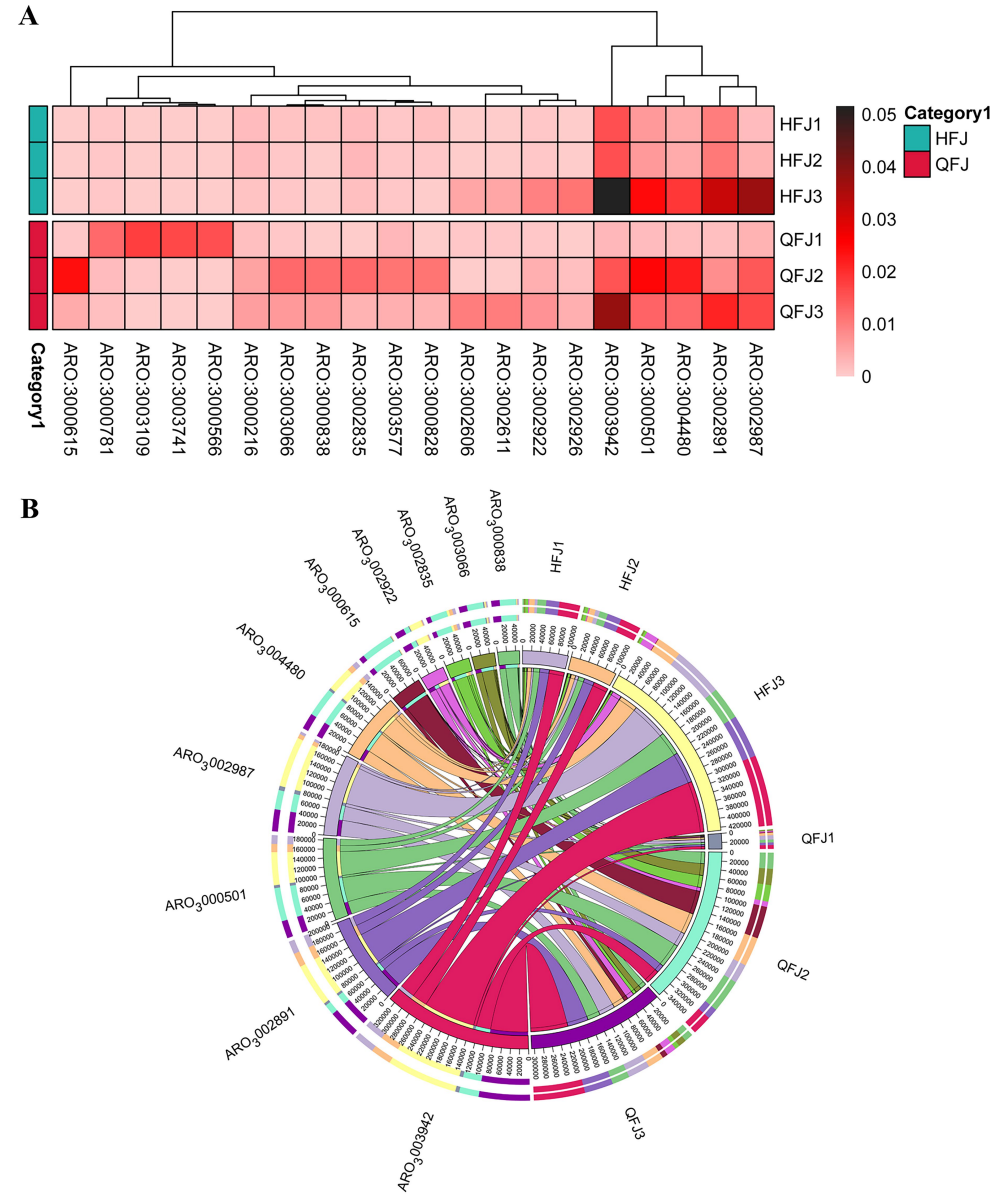
Resistome landscape of fungal-rich (HFJ) versus bacteria-rich (QFJ) environments. **(A)** Heatmap of RPKM-normalized abundances for the 20 most abundant antibiotic resistance genes (CARD ARO IDs). Color intensity reflects gene load; hierarchical clustering shows that QFJ libraries harbor a distinct, ARG-enriched profile. Additional information, such as corresponding gene names, resistance mechanisms, and drug classes, is included in Supplementary Table S5. **(B)** Chord diagram linking individual libraries (outer ring) to detected ARG families (inner labels). Ribbon width is proportional to read counts, illustrating the greater diversity and copy number of *β*-lactamase, aminoglycoside- and tetracycline-resistance genes in QFJ compared with HFJ.

Moreover, hierarchical clustering based on ARG abundance patterns, visualized in the heatmap and phylogenetic tree (Figures 4A,B), revealed a distinct separation between HFJ and QFJ samples. Such clustering reflects the influence of microbial community composition and ecological context on resistome structure, as previously shown in studies of soil resistomes across environmental gradients (Forsberg et al., 2014). These findings underscore the divergent resistance landscapes in fungal-rich versus bacterial-enriched environments.

### 3.5 Comparative analysis and statistical significance

Comparative analyses revealed significant differences in microbial diversity and community composition between the HFJ and QFJ groups. Alpha diversity, measured using the Shannon index, was higher in QFJ samples, consistent with their more complex bacterial composition—a metric widely used to evaluate species richness and evenness in microbiome studies (Forsberg et al., 2014). Beta diversity, assessed through Principal Coordinate Analysis (PCoA) based on Bray–Curtis dissimilarity, demonstrated a distinct separation between HFJ and QFJ groups along the primary axis (PC1), suggesting divergent community structures (Lozupone and Knight, 2005).

In the HFJ samples, *Saccharomyces cerevisiae* and Fructilactobacillus fructivorans were significantly overrepresented (*p* < 0.01), while QFJ samples were enriched in *Acinetobacter soli* and *Klebsiella pneumoniae* (*p* < 0.05). DESeq2 is well-established for such differential abundance analysis, offering robust modeling of microbiome count data. Functionally, genes associated with carbohydrate metabolism were enriched in HFJ (*p* < 0.01), reflecting fungal fermentation dominance, while lipid and amino acid metabolism pathways were more prominent in QFJ (*p* < 0.05), consistent with their diverse bacterial taxa. Additionally, ARGs conferring resistance to beta-lactams and aminoglycosides were significantly more abundant in QFJ (*p* < 0.05), mirroring broader environmental trends (Napit et al., 2025).

## 4 Discussion

This study highlights the effectiveness of metagenomic approaches for analyzing microbial communities within fungal-rich environments. Metagenomics enables high-throughput, culture-independent profiling of microbial communities, revealing their taxonomic structure, metabolic functions, and resistance gene content directly from environmental DNA (Brakhage, 2013). Direct evaluation of the genetic material extracted from environmental samples enabled us to circumvent the limitation of traditional culture-dependent methods. This yielded detailed taxonomic, functional, and resistance profiles that more accurately reflect the microbial ecology of the studied systems. The analysis revealed distinct microbial structures between the HFJ and QFJ groups, with significant differences in dominant taxa, functional pathways, and antibiotic resistance gene (ARG) profiles. These findings underscore the importance of fungal-dominated niches in shaping microbial community dynamics and the dissemination of antibiotic resistance, particularly through ecological interactions that may facilitate horizontal gene transfer (Nazir et al., 2017).

Taxonomic annotation revealed a significant contrast between the two experimental groups. The HFJ group was dominated by eukaryotic organisms, particularly *Saccharomyces cerevisiae*, which accounted for over 85% of the community composition. This pattern aligns with studies demonstrating that *S. cerevisiae* can dominate under certain environmental or enrichment conditions, particularly in nutrient-rich, fermentative niches such as fruit surfaces and controlled fermentation systems (Mortimer and Polsinelli, 1999). In contrast, the QFJ samples exhibited greater bacterial representation, with phyla such as *Firmicutes* and *Proteobacteria* comprising up to 32.7% of the microbial community. This shift reflects the well-documented ecological versatility of these phyla in colonizing diverse environmental matrices and contributing to core microbial functions (Salvetti et al., 2018). The divergence in community structure suggests that the QFJ group may originate from or be influenced by a distinct ecological environment that favors bacterial colonization and expansion. At the species level, notable differences emerged as well, with *Fructilactobacillus fructivorans* enriched in HFJ and *Acinetobacter soli* observed at higher levels in QFJ1. The presence of *F. fructivorans* is in line with findings from fermentation-based ecosystems where this species contributes to microbial succession and metabolic output (Góngora et al., 2024). Similarly, the detection of *A. soli* in QFJ1 reflects its environmental adaptability, as supported by metagenomic analyses identifying *Acinetobacter* spp. in diverse habitats, including clinical and soil-based settings (Akter et al., 2021).

Functional profiling based on KEGG annotations further supported the distinct ecological niches occupied by the HFJ and QFJ microbial communities (Kanehisa and Goto, 2000). Genes involved in carbohydrate metabolism, including glycolysis and the pentose phosphate pathway, were more abundant in HFJ samples, consistent with the dominance of fermentative yeasts such as *S. cerevisiae*. Beyond *S. cerevisiae*, non-conventional yeasts in HFJ may contribute to metabolic diversification through additional functions such as pectin degradation (e.g., in *Hanseniaspora*) or acetate ester production (e.g., in *Pichia*), potentially indirectly shaping bacterial niche partitioning and ARG accessibility (Curiel et al., 2017). The glycolysis pathway in *S. cerevisiae* facilitates the conversion of glucose to pyruvate, generating ATP and NADH, essential for energy production under anaerobic conditions. Concurrently, the pentose phosphate pathway operates to produce NADPH and ribose-5-phosphate, supporting biosynthetic processes and redox balance. In contrast, QFJ samples displayed enrichment in genes associated with amino acid metabolism and environmental adaptation. Specific pathways such as palmitoleate biosynthesis and fatty acid elongation were more prominent in the QFJ group, indicating a potential metabolic shift toward lipid biosynthesis and adaptation-related functions. The fatty acid biosynthesis pathway initiates the formation of saturated fatty acids from acetyl-CoA and malonyl-CoA, while the elongation pathway extends these fatty acids to produce very long-chain fatty acids, which are integral to membrane fluidity and function. These functional distinctions suggest that the two microbial communities have adapted to different ecological conditions, with HFJ favoring carbohydrate metabolism and QFJ displaying a broader metabolic versatility.

Several non-*Saccharomyces* yeasts and filamentous fungi detected in our HFJ samples possess traits essential for rice-wine fermentation. For instance, *Pichia kudriavzevii* can tolerate extremely low pH and high sugar conditions and contributes fruity and floral esters and higher alcohols to fermented beverages (Mu et al., 2025). Its presence in HFJ likely enhances aroma complexity and may indirectly influence bacterial colonization by producing organic acids. Filamentous fungi such as *Rhizopus arrhizus* are also key to traditional fermentation; strains isolated from rice-wine starter cultures exhibit strong saccharification and fermentation abilities, producing high levels of *α*-amylase, glucoamylase and protease and growing optimally around 32 °C and pH 6.5. These enzymes hydrolyse starch into fermentable sugars that fuel yeast metabolism and shape the metabolic milieu. Co-culturing *Rhizopus* with yeasts has been shown to increase the diversity of flavor compounds in rice wine, including esters, higher alcohols and organic acids (Yuan et al., 2024). Highlighting the roles of these non-conventional fungi underscores the ecological complexity of fermentation microbiomes and suggests that ARG dynamics may depend not only on bacterial abundance but also on the metabolic activities of yeasts and molds.

Glycolysis and the pentose phosphate pathway in *Saccharomyces cerevisiae* were enriched in HFJ samples, and the resistome analysis uncovered notable differences in the abundance and diversity of antibiotic resistance genes (ARGs) between the HFJ and QFJ samples. QFJ samples exhibited higher levels of ARGs, particularly genes conferring resistance to beta-lactams, aminoglycosides, and tetracyclines. This observation aligns with findings from Zhang et al., who reported that these ARG subtypes are among the most prevalent across human, animal, and environmental samples, underscoring their widespread distribution in diverse ecosystems (Zhang et al., 2022). This pattern corresponds with the increased bacterial abundance in QFJ and suggests a greater potential for horizontal gene transfer and resistance dissemination within these communities. Notably, comparable ARG enrichment occurs in fermented sausage microbiomes, where bacterial diversity facilitates plasmid-mediated resistance transfer (Wang et al., 2022). Hierarchical clustering based on ARG profiles separated the HFJ and QFJ groups, further highlighting their divergent resistance landscapes. While the influence of fungal activity on ARG dynamics was not experimentally dissected in this study, the fungal dominance observed in HFJ samples and the associated lower ARG abundance leads us to speculate that fungi might play a modulatory role, potentially through selective suppression or reduced bacterial mobility. This hypothesis is supported by research demonstrating that fungal networks can serve as ecological routes for the enrichment and dissemination of ARGs in soil environments, indicating that fungi may influence ARG dynamics in microbial communities (Zhang et al., 2014). however, whether this influence manifests as suppression or facilitation likely depends on specific ecological context and requires direct experimental validation.

Microbial diversity metrics corroborate the compositional and functional trends observed. The Shannon diversity index, a measure of species richness and evenness, was higher in QFJ samples, indicating greater microbial diversity, likely attributable to the increased bacterial representation. This aligns with findings from Wu et al., who utilized the Shannon index to assess microbial diversity across various body habitats, demonstrating its effectiveness in capturing diversity nuances (Wu et al., 2020). Principal Coordinate Analysis (PCoA) based on Bray–Curtis dissimilarity revealed distinct clustering between HFJ and QFJ groups along the primary axis, supporting the presence of fundamentally different community structures. The Bray-Curtis index is a robust metric for evaluating compositional dissimilarity between microbial communities, effectively visualized through PCoA to discern patterns in complex datasets. Differential abundance analysis using DESeq2 identified key taxa and pathways significantly enriched in each group. For instance, *S. cerevisiae* and *F. fructivorans* were significantly overrepresented in HFJ, while *A. soli* and *K. pneumoniae* were enriched in QFJ. At the functional level, carbohydrate metabolism was significantly enriched in HFJ, whereas lipid and amino acid metabolism pathways were more prominent in QFJ. These differences confirm that the observed taxonomic patterns translate into functional and resistance-level distinctions. The application of DESeq2 in this context is consistent with its documented utility in microbiome studies, where it has been employed to discern differential abundance patterns, taking into account data-specific characteristics to ensure robust and reliable results (Weiss et al., 2017). In addition, the higher bacterial diversity in QFJ sample and the dominance of lipid/aminoacid metabolic pathways further highlight how habitat structure and function play roles in shaping the repertoires of genes and determining the bacterial capability in environment manipulation (Garcia-Garcera and Rocha, 2020).

The identification of distinct ARG profiles and community structures across fungal-rich environments highlights the importance of metagenomic surveillance in such ecosystems. Metagenomic analysis has proven to be a powerful and scalable approach for monitoring antimicrobial resistance genes (ARGs) across diverse environmental matrices, offering a means to track emerging resistance threats globally (Van Goethem et al., 2021; Wu et al., 2024). Fungal-dominated habitats, as shown in this study, might serve either as suppressive buffers or as facilitators of ARG dissemination, depending on their ecological context. This dual role is conceptually supported by evidence showing that fungal hyphal networks can both enhance and inhibit the spread of resistance genes, depending on interactions with microbial consortia (Ruan et al., 2022). In addition, in a meta-analysis of traditional fermented food, a higher content of beneficial bacteria and lower ARG was observed, further advocating for their beneficial health outcomes (Xu et al., 2022). These findings contribute to a growing body of literature emphasizing that environmental reservoirs of resistance—especially in soils and water—must be closely monitored to mitigate potential risks to public health (Berendonk et al., 2015). By profiling microbial communities and resistance genes directly from environmental samples, this study provides critical insights into the structure–function relationships of microbial ecosystems and lays a foundation for developing targeted interventions in antimicrobial resistance management.

Importantly, many reads in our datasets could not be assigned to cultured species, underscoring that a large fraction of the fermentation microbiome remains uncultivated. Shotgun metagenomics enables recovery of DNA from these elusive microorganisms and reveals their metabolic potential and resistance genes (Devanthi and Gkatzionis, 2019). Given that more than 99% of microbial species are estimated to be uncultured (Ariaeenejad et al., 2024), such organisms likely contribute to community function and may act as reservoirs for novel ARGs. Future studies coupling metagenomics with single-cell genomics and targeted cultivation will help clarify the roles of these uncultured taxa in fermentation processes and resistome dynamics.

## 5 Limitations

First, the study includes only six samples (three per fermentation mode). Although such a design is common in exploratory metagenomic surveys due to cost and logistical constraints, the small sample size limits statistical power and generalizability. In addition, although the metagenomic pipeline employed in this study enabled comprehensive characterization of the microbial communities, certain limitations warrant consideration. The absence of detailed metadata regarding the origin or environmental conditions of the HFJ and QFJ samples, such as soil physicochemical properties, geographic coordinates, vegetation cover, and microclimatic conditions, limits the interpretation of ecological drivers behind observed taxonomic and functional shifts. Without this contextual information, interpretations regarding selective pressures, microbial interactions, or habitat-specific ARG proliferation remain largely inferential, as emphasized in recent reviews on environmental metagenomics. Additionally, despite rigorous quality control measurements, including adapter trimming, low-quality read removal, and file integrity verification using MD5 checksums, the complexity of microbial assemblies in high-diversity environments may challenge assembly accuracy and downstream annotation. This challenge is compounded by the fragmented nature of metagenomic sequences, which complicates genome assembly, as traditional assemblers often struggle with the high diversity and uneven coverage inherent in such data. In particular, high-diversity communities are prone to fragmented contigs, chimeric assemblies, and underrepresentation of low-abundance or highly variable genes. These limitations may reduce annotation efficiency and bias the detection of horizontally transferred elements such as ARGs, as highlighted in benchmarking studies of metagenomic assemblers (Sczyrba et al., 2017). Finally, the study’s cross-sectional design precludes inference about temporal variation or causality in the relationship between fungal community dynamics and ARG dissemination. Critically, while the potential influence of fungi on modulating resistome structure is discussed conceptually (e.g., fungal suppression hypothesis), this role was not directly tested through functional assays or metatranscriptomic validation. Therefore, ecological interpretations regarding fungal suppression of bacteria or ARGs remain speculative and should be considered hypotheses requiring direct experimental confirmation Future studies incorporating long-read sequencing technologies, such as those offered by PacBio and Oxford Nanopore, could alleviate these issues by providing longer reads that facilitate more accurate genome assembly and functional annotation. Moreover, integrating metatranscriptomic approaches would offer insights into the active functional profiles of microbial communities, aiding in the elucidation of ecological roles and resistance mechanisms within fungi-associated microbiomes (Shakya et al., 2019). Controlled experiments exploring fungal-bacterial interactions may also elucidate the extent to which fungi influence ARG dynamics *in situ* and test the hypotheses generated here. In particular, the limited number of samples reduces the ability to detect subtle differences in the abundance of antibiotic resistance genes between groups and may contribute to false negatives or inflated effect sizes.

## 6 Conclusion

Metagenomic sequencing of microbial communities in fungal-rich and bacterial-rich fermentation environments revealed distinct taxonomic, metabolic and resistome profiles. HFJ samples, dominated by fungi such as *Saccharomyces cerevisiae*, harbored lower bacterial diversity and were enriched in carbohydrate metabolism pathways. In contrast, QFJ samples exhibited greater bacterial diversity (e.g., Firmicutes and Proteobacteria), enhanced lipid and amino acid metabolic functions and a higher abundance of ARGs (notably beta-lactams, aminoglycosides and tetracyclines). These correlations suggest that fungal dominance may be associated with reduced ARG prevalence, whereas bacterial dominance may facilitate ARG persistence; however, causality cannot be inferred from cross-sectional data. The hypothesis that fungal-rich ecosystems suppress bacterial growth or ARG dissemination remains to be tested experimentally. We therefore advocate for functional assays and longitudinal studies to investigate fungus–bacterium–ARG interactions and to validate whether the patterns observed here reflect ecological mechanisms or sampling variance.

## Data Availability

The original contributions presented in the study are publicly available. This data can be found here: https://doi.org/10.6084/m9.figshare.30689663.
