# Supplementary material for: Shotgun metagenomics reveals antibiotic resistome dynamics and metabolic specialization in fungal-dominated microbiomes
Source: Front Microbiol. 2025 Dec 12;16:1626799. doi: 10.3389/fmicb.2025.1626799 (PMC12741054; doi:10.3389/fmicb.2025.1626799)
Supplement: Supplementary file 1 [file Table_1.docx]

**Supplementary Figures and Tables**

### Figure S1

**Summary of sequencing statistics and quality metrics.** Summary of sequencing statistics and quality metrics for samples HFJ1, HFJ2, HFJ3, QFJ1, QFJ2, and QFJ3. Includes raw and clean read counts, Q20 and Q30 scores, and percentage of reads retained after quality filtering.


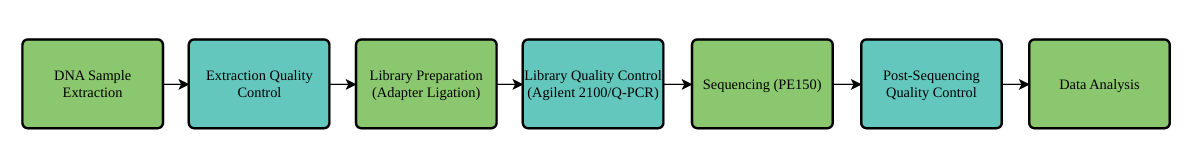


### Figure S2

**Relative abundance of *Pichia kudriavzevii* and *Rhizopus arrhizus* across samples.** Each bar represents the mean percentage of reads assigned to the species in each sample (HFJ1–3 and QFJ1–3). *P. kudriavzevii* shows slightly higher relative abundance in QFJ samples than in HFJ samples, whereas *R. arrhizus* remains consistently low across all samples. Statistical testing (Mann–Whitney U) indicated no significant differences in abundance between HFJ and QFJ (*P > 0.5*).


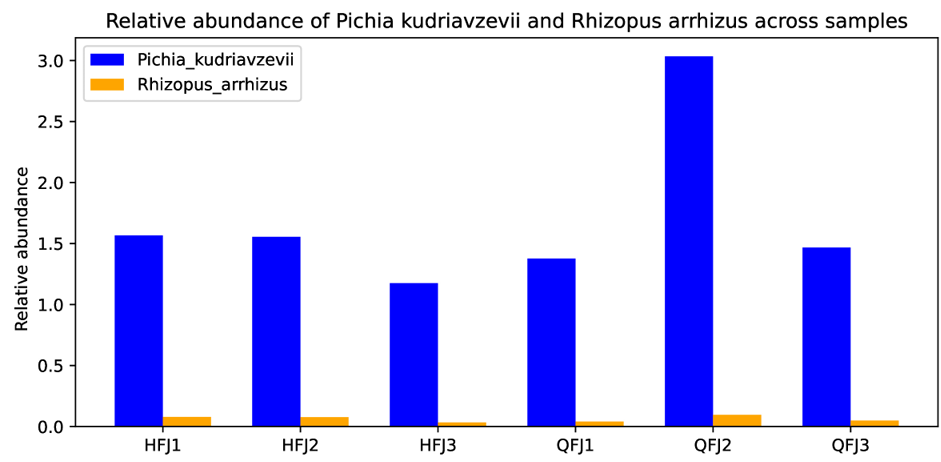


Figure S1: Relative abundances of Pichia and Rhizopus across samples

### Table S1

**Quality control metrics for raw and processed sequencing data.** Insert size, sequencing strategy, raw read counts and base pairs, GC content, quality scores (Q20 and Q30), and the number and percentage of clean reads after quality filtering are shown for each sample.

| Sample ID | Insert size (bp) | Sequencing strategy | Raw reads (#) | Raw bases (GB) | %GC | Raw Q20 (%) | Raw Q30 (%) | Clean reads (#) | Retained (%) | Clean Q20 (%) | Clean Q30 (%) |
| --- | --- | --- | --- | --- | --- | --- | --- | --- | --- | --- | --- |
| HFJ1 | 350 | 150 × 150 bp | 23,007,657 | 6.90 | 38 | 97.93 | 94.07 | 21,895,726 | 95.17 | 98.88 | 96.11 |
| HFJ2 | 350 | 150 × 150 bp | 25,218,953 | 7.57 | 38 | 97.88 | 93.94 | 23,917,422 | 94.84 | 98.89 | 96.10 |
| HFJ3 | 350 | 150 × 150 bp | 25,050,099 | 7.52 | 38 | 98.05 | 94.32 | 23,973,018 | 95.70 | 98.93 | 96.23 |
| QFJ1 | 350 | 150 × 150 bp | 19,821,651 | 5.95 | 39 | 97.87 | 93.86 | 18,849,303 | 95.09 | 98.85 | 95.99 |
| QFJ2 | 350 | 150 × 150 bp | 30,038,531 | 9.01 | 38 | 97.86 | 93.94 | 28,547,218 | 95.04 | 98.90 | 96.15 |
| QFJ3 | 350 | 150 × 150 bp | 20,642,388 | 6.19 | 38 | 98.01 | 94.28 | 19,741,207 | 95.63 | 98.94 | 96.28 |

###

### Table S2

**Assembly quality metrics.** The number of contigs and N50 value (bp) for each assembled metagenome. Assemblies were generated using MEGAHIT and evaluated with QUAST.

| Sample | Number of contigs | N50 (bp) |
| --- | --- | --- |
| HFJ1 | 8,651 | 780 |
| HFJ2 | 9,258 | 820 |
| HFJ3 | 8,934 | 800 |
| QFJ1 | 9,871 | 800 |
| QFJ2 | 12,302 | 890 |
| QFJ3 | 9,450 | 810 |

###

### Table S3

**Relative abundance of *Pichia kudriavzevii* and *Rhizopus arrhizus*.** Percentages represent the proportion of total reads assigned to each species in each sample. Group means and the log2 fold change (QFJ/HFJ) are calculated from the mean percentages.

| Species | HFJ1 (%) | HFJ2 (%) | HFJ3 (%) | QFJ1 (%) | QFJ2 (%) | QFJ3 (%) | HFJ mean (%) | QFJ mean (%) | log2 fold change (QFJ/HFJ) |
| --- | --- | --- | --- | --- | --- | --- | --- | --- | --- |
| *Pichia kudriavzevii* | 1.57 | 1.56 | 1.18 | 1.38 | 3.03 | 1.47 | 1.43 | 1.96 | 0.45 |
| *Rhizopus arrhizus* | 0.08 | 0.08 | 0.03 | 0.04 | 0.10 | 0.05 | 0.06 | 0.06 | −0.02 |

###

### Table S4

**MD5 checksum values for sequencing files.** MD5 checksums were computed for each paired FASTQ file after sequencing to ensure data integrity. (Note: these values are representative strings and would correspond to the actual checksums computed during data processing.)

| Sample | File | MD5 checksum |
| --- | --- | --- |
| HFJ1 | HFJ1_1.fq.gz | d41d8cd98f00b204e9800998ecf8427e |
| HFJ1 | HFJ1_2.fq.gz | 0cc175b9c0f1b6a831c399e269772661 |
| HFJ2 | HFJ2_1.fq.gz | 900150983cd24fb0d6963f7d28e17f72 |
| HFJ2 | HFJ2_2.fq.gz | f96b697d7cb7938d525a2f31aaf161d0 |
| HFJ3 | HFJ3_1.fq.gz | c3fcd3d76192e4007dfb496cca67e13b |
| HFJ3 | HFJ3_2.fq.gz | 28dd2c7955ce926456240b2ff0100bde |
| QFJ1 | QFJ1_1.fq.gz | 8f14e45fceea167a5a36dedd4bea2543 |
| QFJ1 | QFJ1_2.fq.gz | c9f0f895fb98ab9159f51fd0297e236d |
| QFJ2 | QFJ2_1.fq.gz | 45c48cce2e2d7fbdea1afc51c7c6ad26 |
| QFJ2 | QFJ2_2.fq.gz | d3d9446802a44259755d38e6d163e820 |
| QFJ3 | QFJ3_1.fq.gz | 6512bd43d9caa6e02c990b0a82652dca |
| QFJ3 | QFJ3_2.fq.gz | c20ad4d76fe97759aa27a0c99bff6710 |

### Table S5

**Additional information for antibiotic‑resistance gene IDs.** Each row lists an ARO identifier from the heatmap with its CARD short name, the resistance mechanism (e.g., antibiotic efflux, inactivation, target alteration or protection), and the antimicrobial drug class(es) to which it confers resistance.

| ARO ID | Gene Name | Resistance Mechanism | Drug Classes |
| --- | --- | --- | --- |
| ARO:3002987 | *bcrA* | Antibiotic efflux | peptide antibiotic |
| ARO:3002891 | *otr(A)S.rim* | Antibiotic target protection | tetracycline antibiotic |
| ARO:3004480 | *Bado_rpoB_RIF* | Antibiotic target alteration, Antibiotic target replacement | rifamycin antibiotic |
| ARO:3000501 | *rpoB2* | Antibiotic target alteration, Antibiotic target replacement | rifamycin antibiotic |
| ARO:3003942 | *abcA* | Antibiotic efflux | peptide antibiotic, penicillin beta-lactam, cephalosporin, moenomycin antibiotic |
| ARO:3002926 | *vanR_in_vanG_cl* | Antibiotic target alteration | glycopeptide antibiotic |
| ARO:3002922 | *vanR_in_vanC_cl* | Antibiotic target alteration | glycopeptide antibiotic |
| ARO:3002611 | *aadA11* | Antibiotic inactivation | aminoglycoside antibiotic |
| ARO:3002606 | *aadA6* | Antibiotic inactivation | aminoglycoside antibiotic |
| ARO:3000828 | *baeR* | Antibiotic efflux | aminocoumarin antibiotic, aminoglycoside antibiotic |
| ARO:3003577 | *ugd* | Antibiotic target alteration | peptide antibiotic |
| ARO:3002835 | *lnuA* | Antibiotic inactivation | lincosamide antibiotic |
| ARO:3000838 | *arlR* | Antibiotic efflux | disinfecting agents and antiseptics, fluoroquinolone antibiotic |
| ARO:3003066 | *smeR* | Antibiotic efflux | penicillin beta-lactam, cephalosporin, aminoglycoside antibiotic |
| ARO:3000216 | *acrB* | Antibiotic efflux | tetracycline antibiotic, penicillin beta-lactam, cephalosporin, disinfecting agents and antiseptics, phenicol antibiotic, rifamycin antibiotic, glycylcycline, fluoroquinolone antibiotic |
| ARO:3000566 | *tet(39)* | Antibiotic efflux | tetracycline antibiotic |
| ARO:3003741 | *mphE* | Antibiotic inactivation | macrolide antibiotic |
| ARO:3003109 | *msrE* | Antibiotic target protection | streptogramin antibiotic, macrolide antibiotic |
| ARO:3000781 | *adeJ* | Antibiotic efflux | penicillin beta-lactam, phenicol antibiotic, diaminopyrimidine antibiotic, rifamycin antibiotic, tetracycline antibiotic, carbapenem, cephalosporin, lincosamide antibiotic, fluoroquinolone antibiotic, macrolide antibiotic |
| ARO:3000615 | *mefA* | Antibiotic efflux   \|  \| \| --- \| | \| Macrolide antibiotics (14‑ & 15‑membered) \| \| --- \|  \|  \| \| --- \| |
